# Disaster resilience framework indicators for a city’s disaster resilience planning strategy

**DOI:** 10.4102/jamba.v14i1.1264

**Published:** 2022-05-31

**Authors:** Tanja Terblanche, Luiza O. de Sousa, Dewald van Niekerk

**Affiliations:** 1 African Centre for Disaster Studies, Faculty of Natural and Agricultural Science, North-West University, Potchefstroom, South Africa; 2 Community-based Educational Research (COMBER), Department of Geography Education and Environmental Education, Faculty of Education, North-West University, Potchefstroom, South Africa

**Keywords:** city disaster resilience, disaster risk reduction, indicators, sustainable development, sustainable development goals

## Abstract

Determining the level of a city’s disaster resilience and developing a disaster resilience strategy is an important process towards understanding the current and potential future risk of cities. However, the process of determining and mapping the level of urban disaster resilience presents a challenge for the City of Tshwane, as it requires a consolidated and coordinated commitment and collaboration from various role players. This research study developed disaster resilience framework indicators for the City of Tshwane to determine its current disaster resilience and to contribute to its disaster resilience and sustainable development planning strategy. The research assumed a case study design using a qualitative approach to gather data through document analysis and one-on-one interviews. Ten disaster resilience framework indicators were identified as essential indicators in assisting the City of Tshwane with its endeavour to be a disaster resilient city.

## Introduction

The year 2020 marked a turning point in the global battle for sustainable development (and disaster resilience), with cities once again home to a growing majority of the world’s population (UN-Habitat 2020:xv). The responsibility of cities to achieve the sustainable development goals (SDGs) and be disaster resilient is woven throughout a tapestry of agreements, such as the 2030 Agenda for Sustainable Development (UN 2015), Paris Agreement (UNFCC 2015) and the Sendai Framework for Disaster Risk Reduction (UNDRR 2015) amongst others. Each of the agreements collectively forms the backbone of international development policy, recommendations, targets and indicators with local government as the foundational partner in the drive to a more sustainable future (Basu et al. 2013:251; Wahlström, 2017: 336).

In their 2019 World Cities Report, the United Nations Human Settlement Programme (UN-Habitat) reported that cities are consuming land faster than they grow in population because of the increasingly common phenomenon of urban sprawl (UN-Habitat 2020:xvi). The unbridled expansion of urban areas has profound implications on energy consumption, greenhouse gas emissions, climate change and environmental degradation. The speed and scale of urbanisation also bring challenges, such as the uninterrupted provision of basic services and infrastructure (World Bank 2020:1).

As cities continue to grow, there is an urgent need to focus their efforts on resilience to disasters and disturbances that threaten the safety and sustainability of the cities (Weichselgartner & Kelman 2015:249). Both nature-induced and human-made disasters can disrupt everyday life, causing economic loss and infrastructure damages, as well as the injury or loss of human life. Disasters not only create immediate humanitarian crises but also affect the development of a city in the long term (Asprone & Manfredi 2015:96).

Most cities in the world today, in particular those in developing countries, are vulnerable to disaster risks, especially those without disaster resilience structures. Malalgoda, Amaratunga and Haigh (2013:23) argued that any city that has no resilient systems, as well as no resilient communities, is exceptionally vulnerable to disasters. This line of argument has drawn attention to the importance of the resilience of both the urban environments and communities.

To reduce the risk and impact of disasters and increase the safety and well-being of citizens, cities must be more resilient and prepared to address and respond to disruptions. In this context, improving cities’ level of resilience to natural and unnatural hazards is of the utmost importance and requires a holistic approach (Collier et al. 2013:21; Wilkinson 2012:73). Therefore, understanding the demographic and risk profile of the City of Tshwane (CoT) is important from a disaster risk reduction (DRR) and resilience perspective, as it strongly influences the conditions of the social and economic vulnerability of communities exposed to hazards identified in the city.

At present, there is no tool available that guides the CoT to incorporate DRR and SDGs towards a disaster resilient strategy. A framework with indicators to integrate DRR and the SDGs in CoT’s multisectoral disaster planning strategy could enhance its ability to reduce disaster risk and enhance the city’s resilience to disasters (Bello, Bustamante & Pizarro 2021:4).

The importance of this study is that it provides insight into the essence of disaster resilience in a city as an integrated effort of DRR measures and the SDGs in development planning. This study presents disaster resilience framework indicators for a city’s disaster resilience planning strategy to allow a city to determine its resilience to disasters and to assist cities in incorporating DRR measures and the SDGs into its disaster planning strategy.

## Literature review

Sustainable development seeks to combine two goals, namely (1) meeting the needs of the present, (2) without compromising the ability of future generations to meet their needs. Matoso and Jobbins (2016:1) added that ‘the delivery of basic health, education, clean water and sanitation services and social protection (social safety nets to livelihood enhancing programmes) is a critical component of strengthening resilience’.

A general definition of resilience was published by the National Academies Press, as the ability to prepare and plan for, absorb, recover from and more successfully adapt to adverse events (Sharifi 2016:629; Sharifi & Yamagata 2016:115). Another definition was proposed by the UNDRR (2021) as:

The ability of a system, a community or society exposed to hazards to resist, absorb, accommodate to, and recover from the effect of a hazard in a timely and efficient manner including through the preservation and restoration of its essential basic structures and functions.

Resilience is therefore a process or a constant adaptive capability to adapt to change and proper. Increasing adaptive capability is considered a means of both increasing resilience and decreasing vulnerability (Engle 2011:647; Romero-Lankao et al. 2016:342).

Similar to resilience, sustainable development can also be seen as a process, in addition to a normative state, and can require iterative steps of assessment, planning, monitoring and re-assessment to achieve desired long-term goals. These goals are linked to system integrity, livelihood sufficiency, opportunity, resource maintenance and adaptation (Adger et al. 2005:399; Falk 2013:13), wherein sustainable development is defined as development that meets the needs of the present without compromising the ability of future generations to meet their own needs. Sustainable development requires meeting the basic needs of all and extending to all the opportunities to satisfy their aspirations for a better life (Kakar et al. 2021:14; Pohoată et al. 2020:33; WCED 1987:43–44).

In 2015, UN members agreed on a specific resilience goal for urban areas, as part of the 2030 Agenda for Sustainable Development’s 17 SDGs (UN 2015:1). Within SDG 11 emphasised cities and human settlements adopting and implementing integrated policies and plans towards safety, inclusion, resource efficiency, mitigation and adaptation to climate change and resilience to disasters. Sustainable development goal 11 brings sustainability and resilience to the foreground: ‘Making cities and human settlements inclusive, safe, resilient and sustainable’ (UN 2015:14).

Apart from SDG 11, resilience in cities is acknowledged both explicitly and implicitly in other SDG targets. The SDG 1’s Target 1.5 aims for instance, by 2030, to build the resilience of the poor and those in vulnerable situations and reduce their exposure and vulnerability to extreme climate-related events and other economic, social, and environmental shocks and disasters (UN 2015:15). The SDG 9’s Target 9.1 emphasises building resilient infrastructure (UN 2015:20) whilst SDG 13’s Target 13.1 aims to strengthen resilience and adaptive capacity to climate-related hazards and ‘natural disasters’ (UN 2015:23). Reducing disaster risk and building resilience is therefore an interrelated thrust of the 2030 Agenda for Sustainable Development with cities being identified as one of the key stakeholders in the process of making cities resilient. As such, there is widespread agreement within the literature that local governments have a vital role in making cities resilient to disasters and also achieving the 2030 SDGs (Malalgoda et al. 2013:ii; Schofield & Twigg 2019:3).

However, local government and its practitioners, who have the responsibility to build city resilience, need support and guidance to operationalise the resilience-building process (Weichselgartner & Kelman 2014:249). Cities should be provided with means to determine their resilience and be prepared to continue providing essential services should a disaster incident occur (Sharifi 2016:629). Moreover, the SDGs and their targets stress the need for a framework of indicators to allow cities to determine their resilience and sustainability (Croese, Green & Morgan 2020:5). Strategic measures for monitoring and reporting progress made by cities in their endeavour to be disaster resilient are therefore core elements of disaster risk management and sustainable development (Bello et al. 2021:49; Marzi et al. 2019:3). Rockefeller and Arup (2015) agreed and added that:

[*I*]f governments, donors, investors, policymakers, and the private sector are to foster more resilient cities, they need to understand the factors that contribute positively (or negatively) to resilience at a city scale. (p. 3)

Cities also need to understand the dynamic networks of control and influence that reach beyond a city’s administrative boundary and influence their ability to take appropriate action.

Whilst disaster resilience and sustainable development are a national and provincial government competency in South Africa, their success depends on a city’s systems and their interaction in the development of disaster resilience framework indicators that guide DRR measures and the SDGs within the city’s disaster resilience planning strategy. This research was therefore grounded on systems theory (Von Bertalanffy 1972:415) and complex adaptive systems theory (Railsback 2001:4) as its theoretical frame of reference. Systems theory explores the interaction between system components that would lead to the emergence of a city’s disaster resilience planning strategy (Coetzee 2016:69). The complex adaptive systems theory could therefore contribute significantly to the understanding of how the COT’s departments operate and how decisions at a departmental level impact, positively or negatively, larger system dynamics of the city.

Cities are complex systems that consist of highly diverse and interconnected sub-systems and dependent entities connected by non-linear and multiple interactions. When the complex system is disrupted, it stops operating in normal conditions because of disruption of the interconnections of the sub-systems (Atun 2013:51). Addressing disaster resilience as a complex adaptive system in the CoT emphasised the understanding of individual department capacities and how these departments interact to generate disaster resilience (Hartvigsen, Kinzig & Peterson 1998:427).

A disaster can be regarded as a system that cannot be broken down easily into parts and needs to be analysed as a whole (Salmon et al. 2012:353). Thus, from a systems perspective, disaster management involves mutually dependent systems and processes to prepare, mitigate, prevent, respond and recover from the disaster to reduce the negative impact and consequences of such a disaster on the city and its community (Fan & Mostafavi 2018:1). Systems approach in disaster management should assist in disaster planning and hazard forecasting. The tools of systems analysis should be able to improve the quality of disaster-related decision making. The systems approach can contribute to the enhancement of disaster behaviour by using systems thinking to build an organisational community around a common vision of DRR. The systems approach can lead to effective pragmatism and preventative disaster risk management policies.

Every city’s approach to building resilience looks different, but common amongst them is a need for resilience monitoring and measurement all along the journey (Flax, Armstrong & Yee 2016:2). The objective of this research article is to present indicators of a city disaster resilience framework developed to allow city departments to understand the city as a system and to consider the connection between the elements in the system (Cutter et al. 2008:34; Shi et al. 2019:426). The remainder of this article details the study area, discusses the data generation and methodology, the empirical findings and the conclusion.

## Research case, design and methodology

The study assumed the format of a single case study, where the CoT was the case, using a qualitative approach to take a deeper look into complexities, relationships and processes as well as to identify important indicators that are necessary for the city’s disaster resilience framework.

### Case study: The City of Tshwane metropolitan municipality

The CoT consists of 107 geographically demarcated wards and has just over 3.3 million residents. The CoT contributes approximately 28% to Gauteng’s gross domestic product (GDP) and 10% to the national GDP, which indicates the significant role the city plays in the countries’ economy. The CoT has a diverse and vital economy, with five main sectors, including community services, finance, trade, manufacturing and transport. Other significant dynamic growth sectors include construction, the green economy and research, innovation and development. According to UN-Habitat (2020:12), the CoT has a critical shortage of affordable housing stock to address the needs of its growing population. The city’s service delivery backlog in sanitation (14.9%), water (1.3%), electricity (7.4%) and refuse removal (14.4%) could influence the city’s level of resilience (CoT 2019:48).

The CoT has approximately 6000 km of natural watercourses, which include 19 river types. Water resources for the CoT consist of a series of dams, rivers, wetlands and groundwater resources. Water quality has been affected by numerous issues, such as acid mine drainage, wastewater from treatment plants, fertiliser and pesticide runoff and water flows regimes (e.g. catchment hardening and increased stormwater flows) (CoT 2019:84; CoT 2020:12).

As an economic urban hub, the CoT experiences ongoing urbanisation because of the migration of people to the administrative capital for better living conditions, education and employment opportunities. According to Magidi and Ahmed (2015:32), the proportion of urban areas in the CoT increased from 12.77% in 1984 to 26.70% in 2015. According to the CoT Regional Spatial Development Framework (CoT 2018:29), the CoT’s urban growth is not the result of planned growth but of the extension of its boundaries to incorporate new areas over time, resulting in a vast and complex sprawled city form.

Notwithstanding the urbanisation or migration to urban areas, a lack of access to land and housing through formal means has resulted in the urban poor resorting to informal processes to address their land and housing needs on their own. This has resulted in many poor people dwelling in informal settlements, erected mainly on illegally occupied land. According to the CoT Housing and Human Settlements Department, there are a total of 227 informal settlements in the city (CoT 2019:92). Residents in many informal settlements still only have access to rudimentary water and sanitation services and infrequent refuse removal and area cleaning.

A disaster risk and vulnerability assessment was conducted in the CoT during the 2019–2020 financial year. The assessment was a comparative assessment of the 2007 and 2018 risk assessments. Recurring hazards identified in all disaster risk assessments include veld fires, flooding, sinkholes, hazardous material incidents, transport incidents, mission-critical failure and special event incidents (CoT 2019:31). These hazards are still present in the city with new additional hazards identified in the 2019–2020 risk assessment that include radiological hazards, crime, epidemics, illegal dumping, civil unrest and water pollution.

The population growth, demand for services and the recurring hazards place a huge strain on the existing infrastructure network and resources required to maintain services and ensure sustainable development in the CoT. As a result, the CoT and its regions face a huge challenge to be able to be disaster resilient and achieve the SDGs.

## Design and methodology

Within a single case study design we used a qualitative approach to take a deeper look into complexities, relationships and processes and to identify important indicators that are necessary for a city’s disaster-resilience framework. Using the interpretivism perspective, this research provided a detailed analysis of how the CoT employees experience and perceive disaster resilience and sustainable development in the CoT. These perceptions shaped how disasters, resilience and sustainable development are interpreted and understood within city structures and the city’s knowledge systems. The sampling technique applied for this research study was purposive sampling because the researcher selected participants who have extensive experience in disaster risk management and sustainable development (De Vos et al. 2005:238; Marshall 1996:523). The selected CoT officials who participated in the research study provided feedback that assisted in achieving the objectives of the study.

### Data generation

Despite Merriam (2009:42) stating that a case study does not claim any particular method of data generation or data analysis, elements of data generation applicable to a case study include the sources of data (participants and documents), methods of data generation (document analysis and interviews) and the organisation of data (Rule & John 2011:59; Yin 2009:120). Data generation techniques applied in this research study aimed to obtain sufficient data to answer the research questions of the study.

The case study research used document analysis and one-on-one interviews to gain understanding of DRR and sustainable development in the CoT. The CoT’s documents and institutional publications such as policies, procedures and reports were assessed and studied and included in the case study. Document analysis was conducted on four international frameworks that provided a broad view of how cities can integrate DRR and SDGs into its methodology to disaster resilience (Table 1). An analysis of the documents was supplemented by the literature review that was undertaken before conducting the one-on-one interviews. Table 1 provides the identified documents and four international frameworks analysed in the research study:

**TABLE 1:** The City of Tshwane’s documents and international frameworks analysed.

Variable	Description
**CoT Document**	
CoT Vision 2055 (CoT 2012)	This document described the CoT’s long-term strategy with service delivery as the main focus
CoT Vision 2030: IDP (CoT 2018)	The IDP provides for the spatial development plan and service delivery commitment to the community of CoT
CoT Climate Action Plan (CoT 2021)	The Climate Action Plan combines climate action and climate adaptation plans
CoT Disaster Risk and Vulnerability Assessment Report (CoT 2019)	A comprehensive report on the findings of the 2019 disaster risk and vulnerability assessment conducted in CoT
CoT Disaster Risk Management Plan (CoT 2007)	The CoT Disaster Management Plan that articulates compliance to the Disaster Management Act in terms of key performance indicators and enablers
**International frameworks**	
United Nations’ Disaster Resilience Scorecard (UNDRR 2017)	The framework provides a set of assessments that will allow local government to monitor and review progress and challenges in the implementation of the Sendai framework for DRR and assess their disaster resilience.
Rockefeller Foundations’ City Resilience Index (Rockefeller & Arup 2014)	The framework forms the basis of a tool that should enable all interested in city resilience to convene around a common understanding of that idea and begin to ‘baseline’ what matters most for making cities more resilient.
UN-Habitats’ City Resilience Profiling Tool (UN-Habitat 2017)	The aim is to support local government and relevant stakeholders to transform urban areas into safer and better places to live and improve their capacity to absorb and rebound quickly from all potential shocks or stresses, leading them towards sustainability.
UN-Habitats’ City Resilience Action Planning Tool (UN-Habitat 2018)	The aim to enable local governments to plan and undertake practical actions to strengthen the resilience of the cities.

DRR, disaster risk reduction.

For the one-on-one interviews, 10 CoT officials have been identified. The interviews aim to yield shared understanding and perspectives from the participants on the CoT’s disaster resilience efforts, DRR and sustainable development measures known in the city. One-on-one interviews with semi-structured interview questions using an interview schedule with open-ended questions were applied in the study. The interview questions aimed at providing an understanding of DRR and sustainable development in the CoT were posed to each participant systematically and consistently (Behr 1998:152).

### Data analysis

This study applied qualitative data analysis methods intending to develop a CoT disaster resilience framework to assist the city in incorporating DRR measures and the SDGs in its disaster resilience planning strategy.

Content analysis was used to analyse the CoT documents so as to understand and interpret the city’s strategy in disaster resilience and sustainable development. The four international city resilience frameworks and the 17 SDGs and the Sendai Framework’s priorities are all analysed through thematic analysis. Thematic analysis was also applied to the data from the one-on-one interviews.

### Ethical considerations

Ethical approval was obtained from the FNASREC at the Potchefstroom Campus of the NWU (approval number NWU-01629-20-A9), and participant information sheets were provided with consent forms signed by participants. Before the interview, the participants were informed that they could stop at any time, and consent was provided by the participants who would allow for the use of audio recording.

## Results and discussion

The findings from CoT case study analysis revealed that the DRR measures and SDGs do form part of the different CoT’s planning strategies. All 17 SDGs are found in the CoT’s five planning strategy documents. The case study research revealed that CoT also incorporated the Sendai Framework’s DRR measures into its planning strategy. The findings show that different CoT planning strategy documents consist of measures to achieve disaster resilience and the SDGs.

It was, however, found that the city does not have a specific disaster management model that could provide for a consolidated strategy to address disaster resilience and to achieve the 2030 SDGs. The consequence of not having a consolidated strategy was evident during the one-on-one interviews. The CoT, maybe unbeknownst to the CoT’s strategic management has segmented measures in place to achieve the 2030 SDGs and to be a disaster-resilient city. It will, therefore, be difficult for the CoT to determine its level of disaster resilience. It will also not be possible to determine the achievement of the 2030 SDGs. There is, however, a need to have one consolidated disaster resilience planning strategy that is a combination of strategic activities required to implement DRR measures and to achieve the 2030 SDGs. Having a framework for implementing DRR measures and the SDGs can provide the CoT with practical guidance on actions needed for its disaster resilience planning strategy. The framework presented aims to assist the CoT to ensure that the DRR measures and SDGs feature in its disaster resilience planning strategy.

A disaster resilience framework intends to guide cities towards optimal resilience and to eliminate complacency whilst reminding authorities and stakeholders that there is always more to be carried out to ensure lasting resilience. As confirmed during the one-on-one interviews with city employees, a disaster resilience framework can assist a city to determine its resilience and develop actions for resilience through the implementation of DRR measures and the SDGs. Apart from developing actions to implement DRR and the SDGs, a disaster resilience framework can assist the city in developing a comprehensive strategic plan to provide a broad picture of what must be achieved, as well as enable the CoT and its departments to monitor and consider the implementation of identified measures to enable it to become a disaster-resilient city. Participants of the one-on-one interviews agreed that CoT’s resilience to disasters can be measured; however, the city requires means of data capturing, evaluation and monitoring of requirements set to be considered disaster resilient.

The research analysis focused on five CoT disaster resilience and sustainable development strategic documents (cf Table 1). These documents were also referred to by the participants in the one-on-one interviews as strategic documents to address disaster resilience and sustainable development in the city. These documents made (in some way or another) reference to the four Sendai Framework priorities (and measures) for disaster resilience, as well as the 17 SDGs. The SDGs and the Sendai Framework for disaster resilience were analysed to discover how SDGs and DRR form part of the CoT’s planning strategy. It was found that from the five documents, four (Vision 2055, Vision 2030 Integrated Development Plan [IDP], Climate Action Plan [CAP] Disaster Risk and Vulnerability Assessment [DR & VA]) include all the SDGs. The four Sendai Framework priorities were referred to in all five documents.

The SDGs and the Sendai Framework priorities are therefore considered in the city’s planning strategies. The consideration of the Sendai Framework priorities is supported by Wahlström (UNDRR 2015:5) in the foreword of the UN Sendai Framework 2015–2030 where it is articulated that:

[*T*]he need for improved understanding of disaster risk in all its dimensions of exposure, vulnerability and hazard characteristics; the strengthening of disaster risk governance, including national platforms; accountability for disaster risk management; preparedness to ‘Build Back Better’.

The intention of the analysis of the four frameworks, SDGs and the Sendai Framework was to identify themes and indicators considered essential in a city’s disaster resilience strategy. Table 2 provides 10 indicators with key factors that were identified in the four frameworks, SDGs and Sendai Framework.

**TABLE 2:** Emerging indicators across international city resilience frameworks, Sustainable Development Goals and Sendai Framework.

Indicator	Key factors	Disaster resilience scorecard (UNDRR 2017)	City Resilience framework (Rockefeller & Arup 2014)	City Resilience profiling tool (UN-Habitat 2017)	City Resilience Action planning tool (UN-Habitat 2018)	Sustainable Development Goals (UN 2015)	Sendai framework (UNDRR 2015)
Governance and legislation	Management and leadership Commitment Organisation Political leadership	Strong leadership and commitment (p. 10)	Critical importance leadership in form of commitment (p. 5)	Role and place of administrators (p. 32)	Urban governance (p. 9)	Institution of global governance (p. 30)	Strengthen disaster risk governance (p. 17)
Financial capacity	Financial capacity Financial resources Economic growth Investments	Understand economic impact of disaster and need for investment (p. 26)	Availability of financial resources and contingency funds (p. 11)	Economy and livelihoods (p. 30)	Urban economy and society (p. 9)	Sustainable economic growth (p. 25)	Investing in DRR (p. 18)
Urban development	Urban development Urban planning Land management	Resilient urban development (p. 22)	Integrated development planning (p. 13)	Urban planning and design (p. 36)	Urban planning and land management (p. 25)	Urban planning and management (p. 14)	Land use and urban planning (p. 17)
Environment and Ecosystem management	Safeguard and protect ecosystem Ecosystem management Environmental quality and planning Climate action plan	Protect and monitor critical ecosystems (p. 42)	Ecosystem management (p. 12)	Ecosystem services (p. 44)	Environmental management (p. 37)	Sustainable management of water and sanitation (p. 22)	Protecting ecosystems (p. 10)
Institutional capacity	Mutual support Education Awareness Stakeholders Commitment Partnerships	Strengthen institutional capacity (p. 30)	Empowered stakeholders (p. 13)	Stakeholder relations (p. 33)	Partnership and civil society (p. 24)	Partnership for sustainable development (p. 30)	Collaboration and partnerships (p. 17)
Social capacity	Strengthen social capacity Social inclusion Social protection Participation Capacity Social vulnerability	Strengthen societal capacity (p. 56)	Access to social protection (p. 42)	Social inclusion and protection (p. 36)	Identify poorest areas (p. 17)	Capacity of local communities (p. 29)	Social capacity (p. 11)
Critical infrastructure	Infrastructure resilience Maintenance Contingency plans	Critical infrastructure systems (p. 64)	Continuity of critical services (p. 12)	Basic infrastructure (p. 36)	Resilient infrastructure (p. 18)	Resilient infrastructure (p. 22)	Critical infrastructure (p. 12)
Basic service delivery	Continuity of basic services Urban elements Safeguard infrastructure Institutional capacity	Basic infrastructure (p. 22)	Safeguard human life and health (p. 10)	Urban elements: Basic infrastructure (p. 36)	Resilient infrastructure and basic infrastructure (p. 28)	Access to affordable services (p. 23)	Non-disruption of basic services (p. 12)
Law enforcement	Law enforcement Crime prevention Justice Peace Policies and programmes	Implement laws and codes (p. 10)	Law enforcement and crime prevention (p. 11)	Criminal justice and law enforcement (p. 41)	Policies and legislation (p. 54)	Peaceful and inclusive communities (p. 30)	Adoption policies and programmes (p. 20)
Disaster risk management	Identify risk Risk reduction Risk management Safe cities Disaster response Disaster preparedness Disaster recovery Disaster reconstruction Awareness and education Risk governance	Identify hazards and exposures (p. 18)	Reduce exposure (p. 12)	Identify shock and stresses (p. 34)	Urban disaster risk management (p. 9)	Cities to be safe, resilient and sustainable (p. 25)	Understand disaster risk (p. 14)

DRR, disaster risk reduction.

### Discussion on indicators identified

The given table provides a synopsis of reference found in the four international city resilience frameworks that guided the identification of 10 city disaster resilience indicators. For the successful implementation of risk reduction measures, the system must ensure organisational buy-in (*governance*) with strong leadership and commitment through stakeholder engagement (*leadership*), awareness and education (*capacity*), risk identification and risk mapping (*disaster risk management*) to identify vulnerable infrastructure (*basic services delivery infrastructure*) and vulnerable areas (*social vulnerability*). The system must confirm its resource *capacity* and identify resources needed (*assets, finances*) to implement risk reduction measures (*investments, planning, policies, law enforcement*) to safeguard current resources (*infrastructure, environment, assets*) and provide the ability to respond to (*capacity, disaster risk management*) and recover from a disaster, whilst Build(ing) Back Better (*financial capacity, contingency planning*).

The following section provides further support to the 10 indicators identified:

### Governance and legislation

According to the United Nations Office for Disaster Risk Reduction (UNDRR 2017:4), ‘a strong governance system is characterised by laws and policies, institutions and coordination mechanisms, strong leadership, clear roles and responsibility, resources, monitoring and accountability set up across all sectors’. Institutional resilience will ensure connectedness of the various units of government at times of disruption, the cost and quality of services delivered in relation to the resources collected from the citizens, the strength of the government’s mandate to act on the citizens’ behalf, government’s capacity to institutionalise and adapt lessons learned and the extent of discretionary authority granted to government officials during a crisis, as well as political fragmentation (Cutter, Burton & Emrich 2010:1).

Governance and legislation have a significant role to play in implementing disaster resilience and sustainable development initiatives in cities and are regarded as an essential and indispensable steering tool in a city. Building adequate governance to guard against disasters requires strong, effective leadership and commitment to manage and plan for disaster resilience and to achieve the SDGs (Godschalk 2003:11). Legal policies, guidelines and strategic frameworks with common rules, well-defined legal mandates and plans under which disaster resilience and sustainable development programmes operates are required from government leadership (Van Zeijl-Rozema et al. 2008:410).

The systems approach aids all levels of government to organise disaster-related information that is crucial for leaders to improve their decision-making (Simonovic 2015:81), especially when implementing their decisions through policies. The development and implementation of policies, guidelines and strategies are essential in a system to provide for a holistic approach that has interdependencies and interconnected services that require a multisectoral integrated approach to disaster resilience and sustainable development (Barnett & Bai 2007:143).

### Financial capacity

As a response to increasing disaster impact, the importance of financial instruments such as climate risk insurance has been highlighted in the different global processes and commitments. The 2013 *World Bank Development Report* for instance placed a major focus on financial planning and the financial cost of DRR inaction in the face of growing disaster risk. Public policy (cities in this instance) should focus on providing adequate financial infrastructure and at the same time, implement supervision of systemic risk of its finances that is prudent but promotes development (World Bank 2014:194). There is also potential for the private sector and other economic actors to contribute to a greater extent to DRR in cities. Disaster risk should be an explicit consideration for investors as investing in urban resilience is key to sustainable development and the lack of financial and technical resources could hold cities back from pursuing a resilient future (GFDRR 2021:2). The city can play a role in supporting risk-aware decisions, thus ensuring that the exposure of capital to hazard-prone areas, and therefore the value of exposed economic assets is reduced, together with encouraging structures to integrate disaster risk into their financial management processes (GAR 2015:1). Djalante et al. (2013:2122) urged urban governments to seek the diversification of their financial resources if they are to adequately deal with complexities and to anticipate uncertain impacts from hazards and climate change.

### Urban development

Integrated development planning and management require the protection and safeguarding of the environment and the ecosystem and should minimise damage to the environment (Eisenbeiss 2016:1). The UN 2030 SDGs require that different levels of government be provided with opportunities to collaborate to ensure inclusive, secure, resilient and sustainable urban development. Sharifi and Yamagata (2016:1654) agreed that it is essential to integrate resilience thinking into urban planning and design to achieve sustainable development.

A disaster-resilient city that wants to achieve the SDGs must have elements of effective land use planning and enforcement of planning regulations in place. Therefore, there is an increasing need to integrate DRR into development, not only for resilience but for sustainability. This integration requires measures to analyse the progress of performance, programmes and strategies and to monitor implementation (Bendimerad 2003:28).

### Environment and ecosystem management

In the preamble to the 2030 Agenda, world leaders affirmed that they are determined to protect the planet from degradation, achievable through sustainable consumption and production, sustainably managing its natural resources and taking urgent action on climate change, so that the planet can support the needs of present and future generations. Sustainable development aims to manage the environment and natural resources and is linked by complex relationships and disturbances (Kates et al. 2001:641). Reduced exposure and fragility indicated by environmental stewardship, appropriate infrastructure, effective land-use planning and enforcement of planning regulations are considered essential by Rockefeller and Arup’s City Resilience Framework (2014:8).

### Institutional capacity

Blaikie et al. (2003:3) saw disasters as unnatural events that are produced and intensified by the process of risk accumulation and, amongst others, limited institutional capacity. Understanding a city’s institutional background regarding risk reduction, building resilience and sustainable development can help detect current gaps in local capacity to coordinate and act towards prevention, mitigation, response and recovery in the case of disasters, as well as identifying the best and most-effective approaches to strengthen relevant institutions to achieve the SDGs (UNDDR 2015:34). The SDGs require individuals and organisations across society to acquire new capacities to integrate all dimensions of sustainable development in their work, partner across sectors and monitor, evaluate and report on efforts in line with SDG targets and indicators.

### Social capacity

Cultivating an environment for social connectedness promotes a culture of mutual help through recognition of the role of cultural heritage and education (UNDRR 2014:34). Hosseini, Barker and Ramirez-Marquez (2016:48) agreed that there is a need to better understand social vulnerability and social resilience to provide for social capacity in a city. Social vulnerability is the result of pre-disaster social factors that create a lack of capacity or capability to prepare for, respond to and recover from emergencies. Social vulnerability includes people who are more likely to suffer disproportionately because of their existing social circumstances, such as those associated with age, gender, race, medical illness, disability, literacy and social isolation – and should be capacitated to deal with disasters (UNDRR 2014:25). Society must have the capacity to adapt in the presence of disturbance. It is therefore essential for the city, in terms of understanding its risk to disasters, to assess the vulnerability and capacity, exposure and possible effect on the community.

### Critical infrastructure

Resilience, in the context of critical infrastructure, is the ability of a critical infrastructure to system to prevent, withstand, recover and adapt from the effects of various hazards. The *Critical Infrastructure Protection Act, Act 8 of 2019* (South Africa 2019) classifies critical infrastructure as those whose:

(a) functioning is essential for the economy, national security, public safety and the continuous provision of basic public services; and (b) the loss, damage, disruption or immobilization of such infrastructure may severely prejudice. (i) the functioning or stability of the Republic; (ii) the public interest regarding safety and the maintenance of law and order; and (iii) national security. (p. 24)

Critical infrastructure systems are one of the defining features of a city that heavily rely upon the continuous provisioning of smooth operations to provide day to day services, enrich living standards and stimulate local economic growth (Gencer, Panda & Amaratunga 2021:127).

According to Lee (2019:6), the management of facilities by local governments means regular maintenance and management of disaster-prone areas and hazardous facilities that are vulnerable to disaster. Governments need to continuously monitor the adequacy of their existing infrastructure, upgrade and maintain them as necessary and build new ones to provide for their increasing populations. The importance of critical infrastructure, at local level, makes it necessary to identify the role of local governments in addressing this issue and to understand the barriers they face in undertaking it.

### Basic service delivery

The first and foremost responsibility of a municipality is to provide basic services to its community. Sustainable development goal 11.1 requires local government to ‘ensure access for all to adequate, safe and affordable housing and basic services and upgrade slums’. The International Organisation for Standardisation (ISO) 37101 categorially states that progress towards sustainable development through maintaining and improving city services is a core component of a resilient city (ISO 2016:3). Matoso and Jobbins (2016:1) agreed: ‘the delivery of basic health, education, clean water and sanitation services and social protection (social safety nets to livelihood enhancing programmes) is a critical component of strengthening resilience’. The basis of the systems thinking approach is to consider the relationship between systems (city departments) and the integration of these relationships to achieve the outcome or objective, in this instance delivery of services to the community.

### Law enforcement

Bello et al.’s (2021:23) planning for DRR of the 2030 Agenda for Sustainable Development study identified law enforcement as essential measures to be included in a governance framework for resilience. The 2030 Agenda for Sustainable Development explicitly affirms that there can be no sustainable development without peace and no peace without sustainable development. It draws together the strands of peace, the rule of law, human rights, development and equality into a comprehensive and forward-looking framework. Through the adoption of the 2030 SDG, the UN recognised the importance of justice and the rule of law within the wider social sustainable development framework. Secure resource access, order, law and stability are all elements identified as the goal of an urban system to provide personal safety and security from natural and man-made hazards.

### Disaster risk management

Disaster risk management involves actions to both reduce disaster risk and to manage the remaining residual risk, to strengthen long-term disaster resilience and to secure sustained progress towards the achievement of a country’s sustainable development (Benson 2016:4, 9). As is made evident by the 2030 Agenda, disaster risk influences several different dimensions of development. Targets included in 9 of the 17 SDGs of the 2030 Agenda are directly related to disaster risk management and many of the other targets allude to the importance of disaster management as a pivotal element in development (Bello et al. 2021:22). Sustainable development goal 11b states that:

By 2030 the number of cities and human settlements adopting and implementing integrated policies and plans towards inclusions, resource efficiency, mitigation and adaptation to climate change, resilience to disasters, and develop and implement, in line with the Sendai Framework for DRR 2015–2030, holistic disaster management at all levels. (UN 2015)

The Sendai Framework priorities on DRR focus on disaster risk management and provide measures for disaster preparedness (understanding risk, risk assessments,) risk management (risk governance), mitigations (investing in DRR), response (disaster preparedness for effective response), recover (recovery, rehabilitation and reconstruction) monitor and evaluate (through regular preparedness, response and recovery exercises). The goal of disaster management in a city is the long-term survival of city systems as the impact of a disaster has the possibility of impacting the city’s ability to provide services to the community. Integrated disaster risk management in a city (as a system) requires the multisectoral acceptance of the importance of all the elements of disaster risk management.

## Conclusion

Sustainable development in cities as articulated in SDG 11: *Sustainable Cities and Communities* encapsulates essential factors, such as governance (adopting and implementing policies and plans towards inclusion), basic service delivery, disaster resilience, disaster risk management, mitigation and adaptation to climate change and the implementation of the Sendai Framework. Furthermore, SDG 11 seeks to provide for the well-being of its community through, for instance, basic needs for survival, security, health, social relations and so forth (Da Silva, Kernaghan & Luque 2012:125; Maslow 1943:370). The 10 indicators identified therefore infused DRR and the SDGs.

This article sets out to present indicators of a disaster resilience framework for a city’s disaster resilience planning strategy to allow a city to determine its resilience to disasters and assist it in incorporating DRR measures and the SDGs into its strategy. The article identified 10 disaster resilience indicators for the CoT disaster resilience framework that should assist the city with its disaster resilience strategy. The indicators are governance and legislation, financial capacity, urban development, environment and ecosystem management, institutional capacity, social capacity, critical infrastructure, basic service delivery, law enforcement and disaster risk management.

For the successful implementation of risk reduction measures, the system must ensure organisational buy-in (governance) with strong leadership and commitment through stakeholder engagement (leadership), awareness and education (capacity), risk identification and risk mapping (disaster risk management) to identify vulnerable infrastructure (basic services delivery infrastructure) and vulnerable areas (social vulnerability). The system must confirm its resource capacity and identify resources needed (assets, finances) to implement risk reduction measures (investments, planning, policies, law enforcement) to safeguard current resources (infrastructure, environment, assets) and provide the ability to respond to (capacity, disaster risk management) and recover from a disaster, whilst Build(ing) Back Better (financial capacity, contingency planning).
